# Establishing risk of vision loss in Leber hereditary optic neuropathy

**DOI:** 10.1016/j.ajhg.2021.09.015

**Published:** 2021-10-19

**Authors:** M. Isabel G. Lopez Sanchez, Lisa S. Kearns, Sandra E. Staffieri, Linda Clarke, Myra B. McGuinness, Wafaa Meteoukki, Sona Samuel, Jonathan B. Ruddle, Celia Chen, Clare L. Fraser, John Harrison, Alex W. Hewitt, Neil Howell, David A. Mackey

**Affiliations:** 1 Centre for Eye Research Australia, Royal Victorian Eye and Ear Hospital, 32 Gisborne Street, East Melbourne, 3002 VIC, Australia; 2 Ophthalmology, University of Melbourne, Department of Surgery, Parkville, 3010 VIC, Australia; 3 Melbourne School of Population and Global Health, University of Melbourne, Melbourne, 3010 VIC, Australia; 4 Flinders Medical Centre, Flinders University, Bedford Park, 5042 SA, Australia; 5 Save Sight Institute, Discipline of Ophthalmology, Faculty of Health and Medicine, The University of Sydney, 2000 NSW, Australia; 6 Department of Ophthalmology, Royal Brisbane and Women’s Hospital, Herston, 4029 QLD, Australia; 7 School of Medicine, Menzies Institute for Medical Research, University of Tasmania, Hobart, 7005 TAS, Australia; 8 Matrilinex LLC, San Diego, CA 92122, USA; 9 Centre for Ophthalmology and Visual Science, Lions Eye Institute, University of Western Australia, 2 Verdun Street, Nedlands, 6009 WA, Australia

**Keywords:** LHON, optic atrophy, penetrance, genetic counseling, blindness, mitochondria, epidemiology, risk, vision loss

## Abstract

We conducted an updated epidemiological study of Leber hereditary optic neuropathy (LHON) in Australia by using registry data to establish the risk of vision loss among different LHON mutations, sex, age at onset, and mitochondrial haplogroup. We identified 96 genetically unrelated LHON pedigrees, including 56 unpublished pedigrees, and updated 40 previously known pedigrees, comprising 620 affected individuals and 4,948 asymptomatic carriers. The minimum prevalence of vision loss due to LHON in Australia in 2020 was one in 68,403 individuals. Although our data confirm some well-established features of LHON, the overall risk of vision loss among those with a LHON mutation was lower than reported previously—17.5% for males and 5.4% for females. Our findings confirm that women, older adults, and younger children are also at risk. Furthermore, we observed a higher incidence of vision loss in children of affected mothers as well as in children of unaffected women with at least one affected brother. Finally, we confirmed our previous report showing a generational fall in prevalence of vision loss among Australian men. Higher reported rates of vision loss in males with a LHON mutation are not supported by our work and other epidemiologic studies. Accurate knowledge of risk is essential for genetic counseling of individuals with LHON mutations. This knowledge could also inform the detection and validation of potential biomarkers and has implications for clinical trials of treatments aimed at preventing vision loss in LHON because an overestimated risk may lead to an underpowered study or a false claim of efficacy.

## Introduction

Leber hereditary optic neuropathy (LHON [MIM: 535000]) is a primary mitochondrial disease characterized by optic atrophy due to degeneration of retinal ganglion cells in the retina.^1^ LHON is caused by point mutations in mitochondrial DNA (mtDNA) genes encoding subunits of oxidative phosphorylation complex I. Most people with vision loss from LHON harbor one of three primary LHON mutations in MT-ND4 (m.11778G>A [p.Arg340His]),^2^ MT-ND6 (m.14484T>C [p.Met64Val]),^3^^,^^4^ or MT-ND1 (m.3460G>A [p.Ala52Thr]).^5^^,^^6^ The presence of a LHON mutation is not in itself sufficient to cause vision loss. Studies have suggested an association between mtDNA haplogroup and risk of vision loss.^7^, ^8^, ^9^ Similarly, some environmental risk factors, including tobacco smoking, heavy alcohol consumption, and exposure to toxic drugs, may trigger vision loss in some LHON mutation carriers.^10^, ^11^, ^12^ Additional genetic risk factors remain unidentified, and it is currently unknown why only some carriers lose vision.^13^

A risk of vision loss of 50% among men who carry a LHON mutation is still widely cited, although this is most likely a remnant from the time when LHON was thought to be an X-linked recessive disease.^14^ Furthermore, asymptomatic men are likely to be missed in pedigree ascertainment, resulting in an over-estimation of the risk of vision loss. In studies with thorough genealogist-supported pedigree ascertainment, a lower risk of vision loss among male mutation carriers has been observed.^15^, ^16^, ^17^ The risk of vision loss for females is lower than for males, and a recent study suggested there is a steady lifetime risk for female carriers rather than a peak risk in early adult life as seen in male carriers.^18^ In addition, some families appear to have markedly higher or lower penetrance or decreased penetrance over successive generations.^19^^,^^20^

Communication about genetic risks is an important component of genetic counseling. The 2006 definition of genetic counseling states that the process integrates “Interpretation of family and medical histories to assess the chance of disease occurrence or recurrence.^21^” Thus more accurate knowledge of risk of LHON-associated vision loss is critical. Genetic counseling promotes informed choices in view of risk assessment, family goals, and ethical and personal values. It also offers support and assists the person with vision loss and their family with effective coping strategies for dealing with increased levels of uncertainty associated with LHON. Vision loss from LHON is catastrophic and with a lack of effective treatments some individuals may consider reproductive options such as donor egg IVF and mitochondrial donation—where legally available—to reduce their risk of having an affected child with LHON. Accurate information on the risk of vision loss would allow families to make informed choices when planning their families. Additionally, planning future clinical trials of treatments aimed at preventing vision loss in LHON will need accurate data on risk and age of vision loss because an inaccurate risk may lead to an underpowered study or a false claim of efficacy.

Here, we conducted an epidemiological and penetrance study of LHON by using a clinical register to cover the entire population of Australia. There are currently 96 LHON families in Australia, 40 of whom had been published in our previous studies.^15^^,^^22^ We aimed to establish the number of affected and asymptomatic individuals for each pedigree to estimate prevalence and penetrance among different LHON mutations, sex, age at onset, and mitochondrial haplogroup. Furthermore, we aimed to determine the risk of vision loss for offspring of affected mothers and among nieces and nephews of affected men. Finally, we investigated whether the rate of vision loss among men from the largest LHON pedigree in Australia had continued to fall.

## Subjects and methods

### Ethics

This study was conducted in accordance with the revised Declaration of Helsinki and following the Australian National Health and Medical Research Council statement of ethical conduct in research involving humans. Ethical approval was obtained from the Royal Victorian Eye and Ear Hospital Human Research Ethics Committee and University of Western Australia Human Research Ethics Committee. Written, informed consent was provided by all individuals who provided blood or saliva samples.

### Participants

A registry of individuals with LHON hosted by the Royal Victorian Eye and Ear Hospital Ocular Diagnostic Clinic (Melbourne) was established in 1994. Individuals seen at this clinic and those referred to Professor David Mackey from ophthalmologists across Australia since 1990 were included on the registry (see detailed supplemental subjects and methods for further details). The presence of a LHON mutation was determined during clinical genetic testing by a National Association of Testing Authorities-accredited laboratory and confirmed for all pedigrees as described below. In the “affected” LHON group, individuals presented with characteristic clinical fundus changes^23^ and a history of painless, acute vision loss in one eye, with the fellow eye either simultaneously or sequentially affected. Visual acuity measured in a logMAR vision chart in affected individuals ranged from 6/60 down to perception of light vision. Asymptomatic LHON mutation carriers (“carriers”) were recruited or identified from maternal lineages of affected individuals (methods for identifying lineage detailed in supplemental subjects and methods). To minimize the likelihood of including carriers who may yet lose vision, we only included carriers over 25 years old in the analysis because this is the average age of vision loss onset in LHON reported in current literature.^16^^,^^24^^,^^25^

### DNA extraction and mtDNA sequencing

Genomic DNA was extracted from peripheral whole blood via a QIAamp DNA Blood Maxi Kit (QIAGEN, Hilden, Germany) or from saliva via an Oragene DNA saliva collection kit (DNA Genotek, Ontario, Canada) according to the manufacturers’ protocols.

The presence of a LHON mutation was confirmed by polymerase chain reaction (PCR; Invitrogen; primers used are included in Table S1) and Sanger sequencing, whole mtDNA sequencing,^26^ or MitoChip high-throughput sequencing microarray,^27^ followed by alignment against the mtDNA revised Cambridge Reference Sequence (GenBank: NC_012920.1). In homoplasmic pedigrees, maternal relatives of an individual carrying a homoplasmic pathogenic mutation are also carriers of the same mutation and therefore not all maternal relatives were tested.

### Mitochondrial haplogroup determination

Mitochondrial haplogroup was determined either by whole mtDNA sequencing or by PCR and Sanger sequencing of the mitochondrial D-loop hypervariable regions 1 and 2 (HV1 and HV2; primers used are included in Table S1). MtDNA haplogroup was obtained by sequence comparison with MitoMaster^28^ or PhyloTree.^29^ Haplogroup-defining variants identified in each pedigree sequenced are shown in Tables S2 and S3. Partial mtDNA control region sequencing resulted in top level haplogroup determination only.

### Statistical analysis

We used number and percent to describe the distribution of mutation type, haplotype, and sex among included individuals. Age of vision loss onset among affected individuals was summarized as median, range, and interquartile range (IQR) and was compared according to sex via the Wilcoxon rank-sum test.

Because mortality data were not available for all participants, individuals aged 85 years and above on December 1, 2020 were excluded from prevalence estimates, as were those known to be deceased. Australian population estimates were derived from the 2016 census data (source: Australian Bureau of Statistics Table-Builder, accessed December 1, 2020, web resources).

Penetrance was estimated with 95% confidence intervals (CIs) as the proportion of LHON mutation carriers who manifest clinically discernible loss of vision. Penetrance was determined in each pedigree independently, and combined penetrance values were calculated for each LHON mutation and for males and females separately. For all penetrance analyses, only asymptomatic carriers over 25 years of age as of December 1, 2020 were included. However, all affected individuals were included (including those deceased or over those 85 years of age).

We used multivariable logistic regression with robust standard errors to account for intra-family correlation to investigate the association between vision loss and mtDNA haplogroup (H, J, other, or unknown), after adjusting for sex and mutation. Only a subgroup of individuals—those carrying the m.11778G>A or m.14484T>C mutations—were included in this analysis because these were the only mutations observed in combination with both J and H haplogroups.

## Results

### Minimum LHON prevalence in Australia

**Figure 1:**
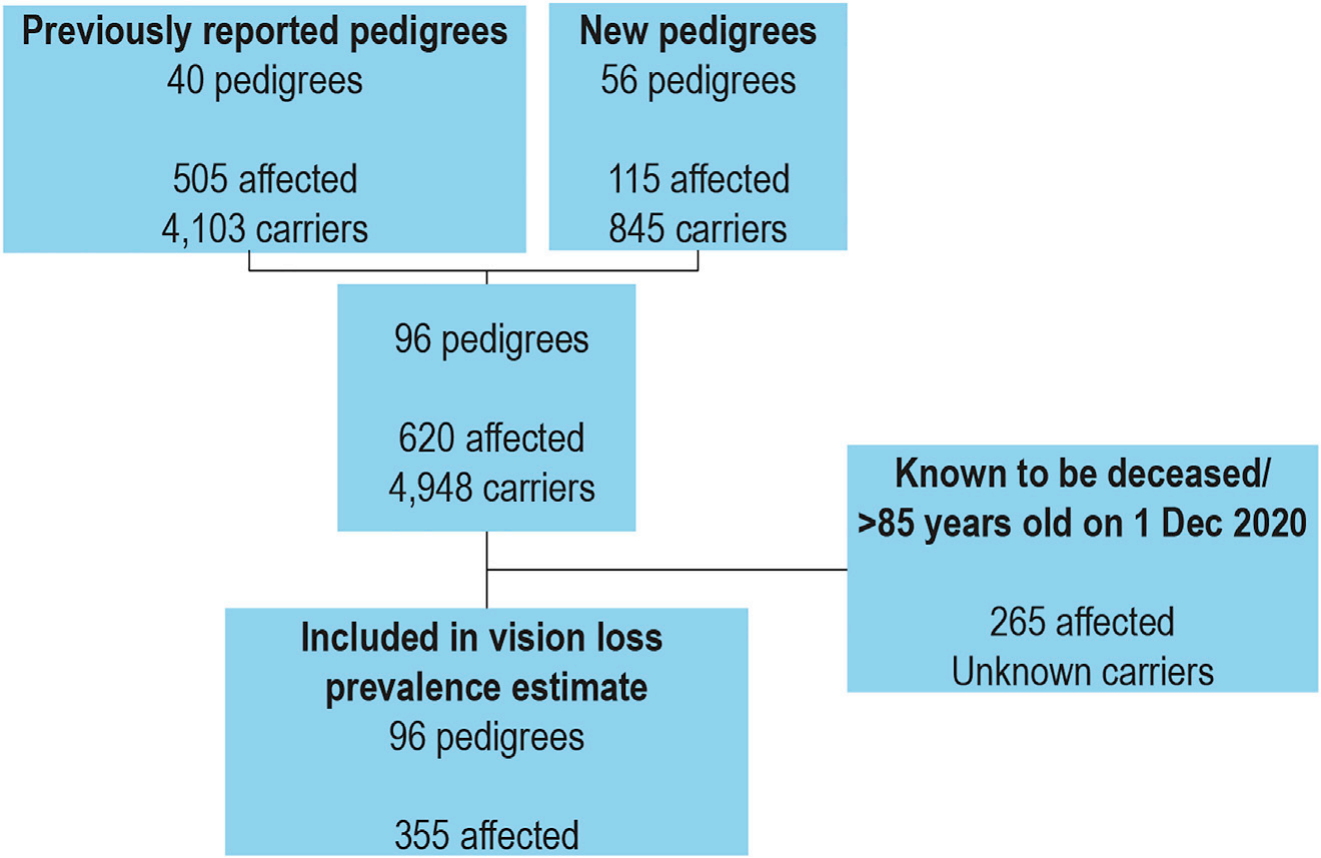
Flow chart of included individuals with LHON Previously published and new pedigrees with numbers of affected and unaffected carriers included in study.

We identified 96 genetically unrelated LHON pedigrees, including 56 new pedigrees, and updated 40 previously known pedigrees in Australia (Table 1).^4^^,^^22^^,^^30^, ^31^, ^32^, ^33^, ^34^, ^35^ Updated assignments of LHON matrilineal pedigrees are shown in Table S4. Among the pedigrees, 43/96 (44.8%) were sporadic cases, with only one affected person identified thus far, and 53/96 (55.2%) were familial. In total, we identified 5,568 individuals harboring LHON mutations, including 620 affected individuals (462 males and 158 females) and 4,948 matrilineal carriers (2,171 males and 2,777 females) (Table 1). After excluding known deceased individuals and people over 85 years old, 355 live individuals remained (Figure 1). This is equivalent to a minimum LHON vision loss prevalence in Australia of one in 68,403 from a population of 22,915,047.

**Table 1:** Individual LHON pedigrees in Australia

	**Pedigree**	**Mutation**	**Affected male**	**Affected female**	**Carrier male**^a^	**Carrier female**^a^	**Family penetrance**^b^	**mtDNA haplogroup**	**Reference**
1	ACT02	11778	1	2	0	3	50%	unknown	new
2	NSW01	11778	8	3	45	59	10%	H1n	Mackey and Buttery^15^
3	NSW02	11778	8	3	68	80	7%	H	Mackey and Buttery^15^
4	NSW03	11778	3	2	26	29	8%	U5a1	Mackey and Buttery^15^
5	NSW04	11778	21	7	55	70	18%	J1c1	Chan et al.^22^
6	NSW05	11778	3	2	13	13	16%	unknown	Chan et al.^22^
7	NSW06	11778	9	5	12	15	34%	H4a1	Chan et al.^22^
8	NSW09	11778	8	6	14	21	28%	J1c4	new
9	NSW11	11778	2	1	4	7	21%	Y2a	new
10	NSW16	11778	1	0	3	3	14%	unknown	new
11	NSW17	11778	1	0	5	5	9%	T2f1	new
12	NSW18	11778	1	0	2	6	11%	J1c1	new
13	NSW20	11778	4	2	3	7	37%	unknown	new
14	NSW23	11778	3	0	4	7	21%	J	new
15	NSW25	11778	1	0	9	10	5%	unknown	new
16	NSW26	11778	0	2	0	5	28%	K	new
17	NZ01	11778	0	1	0	1	50%	U	Chan et al.^22^
18	NZ02	11778	7	3	14	28	19%	U5b2a	Chan et al.^22^
19	NZ03	11778	2	0	0	3	40%	J	Chan et al.^22^
20	NZ04	11778	1	0	1	3	20%	unknown	Chan et al.^22^
21	NZ05	11778	1	0	0	1	50%	unknown	Chan et al.^22^
22	NZ07	11778	1	0	0	3	25%	unknown	new
23	NZ08	11778	1	0	3	8	8%	unknown	new
24	NZ09	11778	3	0	0	2	60%	unknown	new
25	NZ12	11778	0	1	3	2	16%	H	new
26	QLD02	11778	7	2	33	40	11%	J2b1	Mackey and Buttery^15^
27	QLD03	11778	1	0	9	14	4%	J1c2	Chan et al.^22^
28	QLD04	11778	1	0	3	4	12%	T2b4	Chan et al.^22^
29	QLD05	11778	0	1	6	10	6%	HV	new
30	QLD07	11778	1	0	4	9	7%	unknown	new
31	QLD09	11778	0	1	5	5	9%	unknown	new
32	QLD10	11778	1	0	6	9	6%	N	new
33	QLD11	11778	1	0	1	5	14%	W	new
34	QLD12	11778	2	1	1	6	30%	U	new
35	SAU03	11778	1	0	3	8	8%	unknown	new
36	TAS01	11778	112	27	899	1025	7%	H	Mackey and Buttery^15^
37	TAS05	11778	2	0	22	21	4%	H3f	Mackey and Buttery^15^
38	VIC03	11778	7	0	14	23	16%	U	Mackey and Buttery^15^
39	VIC04	11778	6	0	34	40	7%	H1c3	Mackey and Buttery^15^
40	VIC05	11778	1	0	12	20	3%	K2a4	Mackey and Buttery^15^
41	VIC06	11778	7	2	10	14	27%	H1	Mackey and Buttery^15^
42	VIC07	11778	11	0	16	38	17%	I2a	Mackey and Buttery^15^
43	VIC09	11778	1	0	4	6	9%	unknown	Chan et al.^22^
44	VIC10	11778	6	2	8	9	32%	J2a1a	Chan et al.^22^
45	VIC15	11778	2	0	9	9	10%	unknown	new
46	VIC17	11778	1	1	2	2	33%	J1c5a	new
47	VIC18	11778	1	1	2	2	33%	U4c1	new
48	VIC19	11778	1	0	4	8	7%	B4b1a2	Craig et al.^31^
49	VIC21	11778	4	3	24	27	12%	H13a1a	new
50	VIC22	11778	1	0	14	21	3%	H13a1a	new
51	VIC25	11778	1	0	4	5	10%	T1a1	new
52	VIC29	11778	1	1	32	33	3%	U5a1a1	new
53	VIC31	11778	2	0	4	4	20%	J	new
54	VIC39	11778	1	0	3	4	12%	unknown	new
55	VIC40	11778	3	1	3	5	33%	unknown	new
56	VIC41	11778	1	0	0	2	33%	H	new
57	VIC43	11778	1	0	1	3	20%	K	new
58	VIC45	11778	3	0	0	4	42%	unknown	new
59	VIC46	11778	1	0	5	4	10%	B	new
60	WAU01	11778	16	5	42	55	18%	J	Mackey and Buttery^15^
61	WAU02	11778	6	1	20	25	13%	K	Chan et al.^22^
62	WAU03	11778	1	0	21	39	1%	I2a	Chan et al.^22^
63	WAU04	11778	1	1	22	19	4%	H3	new
64	WAU11	11778	1	1	6	19	7%	unknown	new
65	WAU12	11778	2	0	1	6	22%	unknown	new
66	ACT01	14484	3	0	1	12	19%	J	Chan et al.^22^
67	NSW10	14484	0	1	0	1	50%	unknown	new
68	NSW12	14484	1	0	22	44	1%	K1c2	new
69	NSW15	14484	2	0	9	15	8%	U5a1a1	new
70	NSW19	14484	6	1	21	26	13%	L1b1a1	new
71	NSW24	14484	1	0	2	3	17%	unknown	new
72	NZ06	14484	1	0	3	9	8%	unknown	new
73	NZ10^c^	14484	1	0	13	13	4%	J	new
74	NZ11	14484	1	0	5	12	6%	J	new
75	SAU02	14484	1	1	1	1	50%	unknown	Chan et al.^22^
76	SAU05	14484	0	1	5	15	5%	H	new
77	TAS02	14484	74	9	320	380	11%	J1c2c	Mackey and Buttery^15^
78	VIC02^c^	14484	2	4	1	3	60%	J1b1a	Mackey and Buttery^15^
79	VIC08	14484	1	0	7	9	6%	J1c1	Mackey and Buttery^15^
80	VIC11	14484	5	5	12	22	23%	J1c1	Chan et al.^22^
81	VIC14	14484	1	0	17	34	2%	H3c	Howell et al.^26^
82	VIC24	14484	1	0	3	2	17%	unknown	new
83	VIC30^c^	14484	1	0	9	15	4%	H	new
84	VIC42	14484	1	0	7	11	5%	J	new
85	WAU7	14484	1	0	2	2	20%	unknown	new
86	NSW07	3460	1	1	2	6	20%	T	Chan et al.^22^
87	NSW14	3460	0	1	1	1	33%	unknown	new
88	TAS03	3460	3	0	45	39	3%	H5a1	Mackey and Buttery^15^
89	VIC01	3460	6	3	4	11	37%	H	Mackey and Buttery^15^
90	VIC28	3460	3	0	7	7	18%	H	new
91	WAU06	3460	1	0	7	8	6%	M53	new
92	WAU08	3460	2	0	7	10	10%	unknown	new
93	SAU04	4171	1	1	4	6	17%	HV0	new
94	NSW08	14482	5	2	17	30	13%	I1a	Chan et al.^22^
95	VIC20	11778+14484	1	2	15	23	7%	U5a1a1	Howell et al.^33^
96	QLD01	14484+4160	26	35	9	14	73%	U4a1a	Mackey and Buttery^15^
Total			462	158	2,171	2,777			

LHON pedigrees are organized alphabetically and by LHON mutation.

a Only asymptomatic carriers over 25 years of age are included.

b Rounded to nearest whole percentage.

c Heteroplasmic mutation.

The majority of affected individuals (400/620, 64.5%) harbored the m.11778G>A mutation, and the m.14484T>C mutation was the next most common (126/620, 20.3%; Table 2). The m.3460G>A mutation was present in only 3.4% of affected individuals (21/620). Together, the three primary LHON mutations accounted for 88.2% of total LHON cases. Four additional pedigrees harbored either double LHON mutations (VIC20, m.11778G>A + m.14484T>C;^33^ QLD01 m.14484T>C + m.4160T>C^30^) or rare LHON mutations (NSW08, m.14482C>G;^32^ SAU04, m.4171C>A) (Table 2). All pedigrees harbored homoplasmic mutations, except for VIC02,^31^ VIC30, and NZ10, who harbored heteroplasmic mutations (Figure S1).

**Table 2:** Individuals with vision loss attributable to LHON

**LHON mutation**	**Males with vision loss/ total carriers**^a^**[penetrance]**	**Females with vision loss/total carriers**^a^**[penetrance]**	**Total individuals with vision loss/total carriers**^a^**[penetrance]**
m.11778G>A	309/1,902 [16.2%]	91/2,084 [4.3%]	400/3,986 [10.0%]
m.14484T>C	104/564 [18.4%]	22/651 [3.4%]	126/1,215 [10.4%]
m.3460G>A	16/89 [18.0%]	5/87 [5.7%]	21/176 [11.9%]
m.4171C>A	1/5 [20.0%]	1/7 [14.3%]	2/12 [16.7%]
m.14482C>G	5/22 [22.7]	2/32 [6.2%]	7/54 [13.0%]
m.11778G>A/m.14484T>C	1/16 [6.2%]	2/25 [8.0%]	3/41 [7.3%]
m.14484T>C/m.4160T>C	26/35 [74.3%]	35/49 [71.4%]	61/84 [72.6%]
Total	462/2,633 [17.5%]	158/2,935 [5.4%]	620/5,568 [11.1%]

a Only asymptomatic carriers over 25 years of age are included.

We observed the characteristic sex predominance in LHON; there were 462/620 (74.5%) affected males and 158/620 (25.5%) affected females (Table 2), resulting in a male to female ratio of 2.92:1. The ratios of affected males to affected females for each of the primary LHON mutations were 3.4:1 (m.11778G>A), 4.7:1 (m.14484T>C), and 3.2:1 (m.3460G>A).

### Vision loss penetrance due to LHON

The overall penetrance—or proportion of individuals affected by vision loss among all LHON mutation carriers in Australia—was 17.5% for males (462/2,633, 95% CI 16.1, 19.1) and 5.4% for females (158/2,935, 95% CI 4.6, 6.3) (Table 2). This indicates an overall risk of vision loss for 1 in 6 males and 1 in 20 females harboring a LHON mutation.

Penetrance was slightly variable for each of the three primary LHON mutations: m.11778G>A, 16.2% (males) and 4.3% (females); m.14484T>C, 18.4% (males) and 3.4% (females); and m.3460G>A, 18% (males) and 5.7% (females). Furthermore, penetrance was also highly variable among individual pedigrees, ranging between 1%–73% (Table 1).

It must be noted that our data include two previously described large pedigrees (TAS01 and TAS02) that together account for 35.8% (222/620) of total individuals affected with LHON in our cohort (Table 1). Furthermore, highly penetrant pedigrees included the previously described VIC02 family, which had six out of ten carriers affected by vision loss (60% penetrance), and the QLD01 family, which had 61 out of 84 maternal relatives affected by vision loss (72.6% penetrance).^15^^,^^22^^,^^31^^,^^34^^,^^36^ At least nine members of the QLD01 pedigree presented with neurological abnormalities characteristic of “LHON plus” syndrome.^30^

### Vision loss penetrance among mtDNA haplogroups

Mitochondrial haplogroup was determined in 68 out of 96 pedigrees (Table 1), as suitable DNA samples were not available for sequencing in all lineages. This is equivalent to 84.8% of affected individuals (526/620) and 94.5% of asymptomatic carriers (4,675/4,948). Overall, 13 mtDNA haplogroups (J, H, U, K, T, I, HV, B, N, W, Y, L, and M) were identified (Tables 3 and S5). As expected, given the European ancestry of most individuals in our study, a large proportion of lineages belonged to haplogroups J (19/68; 27.9%) and H (18/68; 26.5%). However, non-European mitochondrial haplogroups, including B, M, I, and Y, were also identified, which reflects more recent immigration to Australia of families from non-European countries. Haplotype H was most common among individuals with mutation m.11778G>A (67.7%), whereas haplotype J was more common among individuals with mutation m.14484T>C (76.9%) (Table S5).

**Table 3:** Associations between mtDNA haplogroup and vision loss

	**Number (%) with vision loss**	**Adjusted OR**	**Adjusted 95% CI**	**Adjusted p value**
Total	526/5,201 (10.1%)	–	–	–

**Sex**				

Female	113/2,736 (4.1%)	1.00	–	–
Male	413/2,465 (16.8%)	4.81	[3.75, 6.16]	<0.001

**Mutation**				

11778	400/3,986 (10.0%)	1.00	–	–
14484	126/1,215 (10.4%)	0.57	[0.41, 0.79]	0.001

**Haplotype**				

H	208/2,797 (7.4%)	1.00	–	–
J	197/1,429 (13.8%)	2.85	[2.04, 3.99]	<0.001
Other	73/700 (10.4%)	1.73	[1.06, 2.81]	0.028
Unknown	48/275 (17.5%)	2.92	[1.77, 4.80]	<0.001

Multivariable logistic regression estimated with all listed variables as covariates and robust standard errors to account for intra-family correlation.

The proportion of individuals with vision loss was similar between those with mutation m.11778G>A or m.14484T>C. However, after adjusting for sex and haplogroup, the odds of vision loss were estimated to be almost half among those with mutation m.14484T>C compared to those with m.11778G>A (adjusted odds ratio [OR] 0.57, p = 0.001; Table 3). Furthermore, after adjusting for sex and mutation, individuals with haplogroup J were estimated to have almost three times the odds of vision loss compared to those with haplotype H (OR 2.85, p = 0.001; Table 3).

### Age of onset of vision loss attributable to LHON

**Figure 2:**
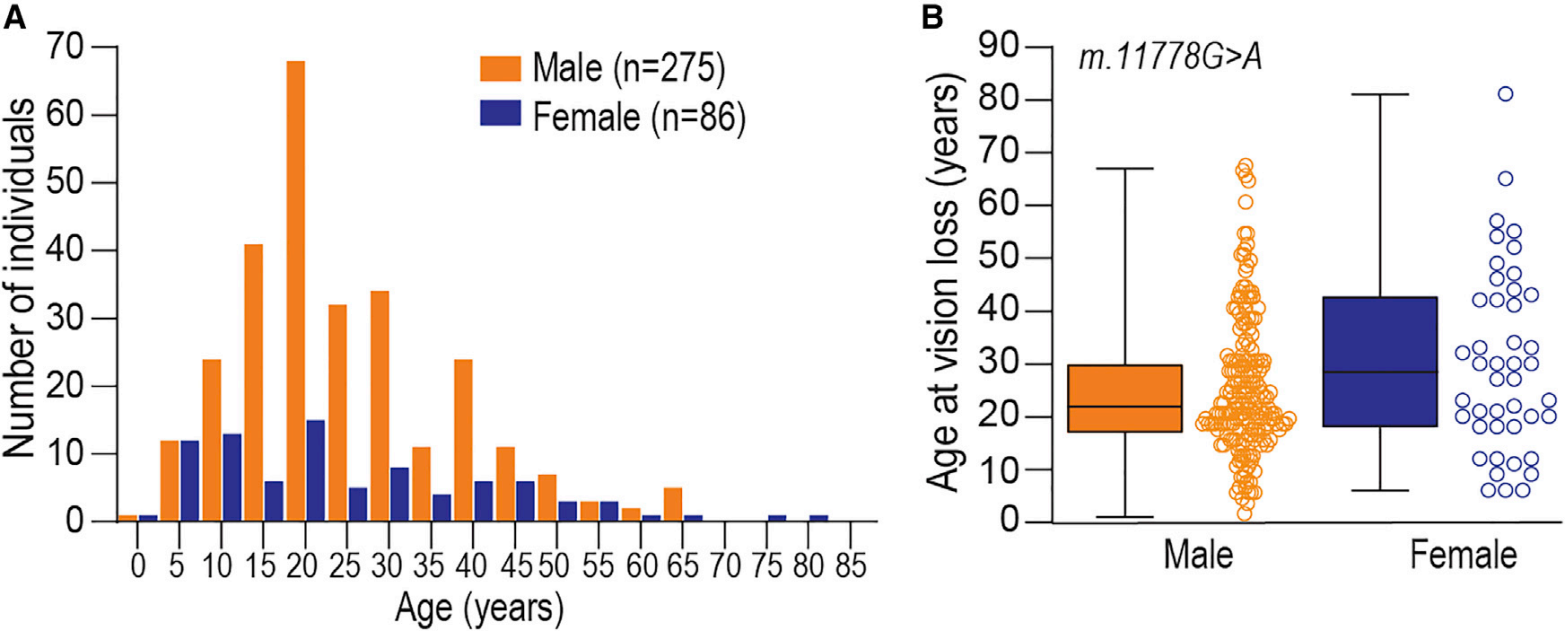
Age of onset of vision loss in individuals harboring LHON mutations (A) Age at onset of vision loss of 361 individuals with LHON mutations. Across all LHON mutations, median age at onset of vision loss was similar between males (22 years, IQR = 17–30; range 1–67) and females (21 years, IQR = 11–39; range 2–81, p value = 0.63, Wilcoxon rank-sum test). (B) Age at onset of vision loss among females (median = 28.5 years; IQR = 18–42; range 6–81) and males (median = 22 years; IQR = 17–67; range 1–67) with the m.11778G>A mutation (p value = 0.07, Wilcoxon rank-sum test).

We were able to ascertain age of vision loss in 361 out of 620 individuals with LHON mutations (Figure 2A). Across all LHON mutations, the median age at onset of vision loss was similar between males (22 years, IQR = 17–30; range 1–67) and females (21 years, IQR = 11–39; range 2–81, p value = 0.63). Age at onset of vision loss was slightly higher for females (median = 28.5 years; IQR = 18–42; range 6–81) compared to males (median = 22 years; IQR = 17–67; range 1–67) with the m.11778G>A mutation (p value = 0.07; Figure 2B). Age of vision loss onset was similar between males (median = 25 years; IQR = 18–37; range 9–65) and females (median = 21 years; IQR = 14–40; range 6–73, p value = 0.55) with the m.14484T>C mutation.

Among males, vision loss onset peaked between 15–24 years of age, accounting for 41.3% (107/259) of male affected individuals harboring one of the three primary LHON mutations. For females, the age at symptom onset was distributed evenly across all ages. Notably, 8.8% (23/259) of males and 10.1% of females (6/59) experienced vision loss at age 10 years or under, while 5.4% of males (14/259) and 11.8% of females (7/59) lost vision age 50 years or over.

### Risk of vision loss in offspring of affected women

It was reported previously that affected women have a higher incidence of affected offspring compared to asymptomatic females.^16^^,^^37^ We updated this analysis to include all Australian LHON pedigrees to date. Among the 158 affected females, 100 had children, 54 did not, and we could not obtain information on the parental status of four women. For this sub-analysis, we excluded four affected females because they were too young to have children (12 years old or younger) and six affected mothers whose children were 22 years or younger and therefore still below the average age of vision loss onset in LHON.

In total, 94 affected mothers and 281 offspring (132 males and 149 females) were included in this sub-analysis. The median number of offspring per mother was 2.5 (IQR = 2–3; range 1–10; Table S6). Across all LHON mutations, affected mothers had 93/281 affected offspring (33.1%, 95% CI 27.6, 38.9), including 49/132 males (37.1%, 95% CI 28.9, 50.0) and 44/149 females (29.5%, 95% CI 22.3, 37.5) (Table 4). This is higher than the overall penetrance of vision loss observed among all LHON mutation carriers in this cohort (17.5% for males and 5.4% for females, Table 2). Importantly, vision loss is twice as likely among offspring of affected mothers with the most common LHON mutation, m.11778G>A (Table 4).

**Table 4:** Vision loss in offspring of affected women with LHON

**Affected mother mutation**	**Males with vision loss/total male offspring**^a^**[penetrance]**	**Females with vision loss/total female offspring**^a^**[penetrance]**	**Total individuals with vision loss/total offspring**^a^**[penetrance]**
m.11778G>A	30/87 [34.5%]	10/91 [11%]	40/178 [22%]
m.14484T>C	3/19 [15.8%]	5/21 [23.8%]	8/40 [20%]
m.3460G>A	0/1 [N/A]	1/3 [33.3%]	1/4 [25%]
m.14484T>C/m.4160T>C	15/24 [62.5%]	27/32 [84.4%]	42/56 [75%]
m.14482C>G	1/1 [100%]	1/2 [50%]	2/3 [66.6%]
Total	49/132 [37.1%]	44/149 [29.5%]	93/281 [33.1%]

N/A, not applicable.

a Only asymptomatic carriers over 25 years of age are included.

Next, we investigated the risk of vision loss in children of an asymptomatic woman with at least one affected brother, focusing on six large LHON pedigrees—TAS01, TAS02, NSW01, NSW02, NSW04, and WAU01 (Table 5). In general, we observed a higher incidence of vision loss in children of women with at least one affected brother (pedigrees TAS01, TAS02, NSW01, and NSW02) compared to the overall incidence of vision loss in each respective pedigree. However, incidence of vision loss was higher only among nieces of affected men in the NSW04 family, and the incidence of vision loss was not increased among nephews or nieces of affected men in the WAU01 pedigree.

**Table 5:** Vision loss in children of asymptomatic women with at least one affected brother in representative LHON families

**Sibship analysis within pedigree**	**Males with vision loss/total male offspring**^a^**[penetrance]**	**Females with vision loss/total female offspring**^a^**[penetrance]**	**Total individuals with vision loss/total offspring**^a^**[penetrance]**
TAS01	48/289 [16.6%]	13/278 [4.7%]	61/575 [10.8%]
*TAS01 (complete)*	*112/1,011 [11.1%]*	*27/1,052 [2.6%]*	*139/2,063 [6.7%]*
TAS02	45/129 [34.9%]	6/149 [4.0%]	51/278 [18.3%]
*TAS02 (complete)*	*74/394 [18.8%]*	*9/389 [2.3%]*	*83/783 [10.6%]*
NSW01	2/9 [22.2%]	1/8 [12.5%]	3/17 [17.6%]
*NSW01 (complete)*	*8/53 [15.1%]*	*3/62 [4.8%]*	*11/115 [9.6%]*
NSW02	3/15 [20.0%]	0/17 [N/A]	3/32 [9.4%]
*NSW02 (complete)*	*8/76 [10.5%]*	*3/83 [3.6%]*	*11/159 [6.9%]*
NSW04	5/33 [15.1%]	6/43 [14.0%]	11/76 [14.5%]
*NSW04 (complete)*	*21/76 [27.6%]*	*7/77 [9.1%]*	*28/153 [18.3%]*
WAU01	5/20 [25.0%]	1/29 [3.4%]	6/49 [12.2%]
*WAU01 (complete)*	*16/58 [27.6%]*	*5/60 [8.3%%]*	*21/118 [17.8%]*

Penetrance for each complete pedigree (from Table 1) is included in italics to facilitate direct comparison.

a Only asymptomatic carriers over 25 years of age are included.

### Decrease in prevalence of vision loss over successive generations

We reported previously an apparent generational fall in rates of vision loss among men from the largest LHON pedigree in Australia, TAS01.^19^ We updated this pedigree with expanded genealogical information and compared it to an earlier publication by Hogg,^38^ which was updated and reported as “pedigree 33” by Hamilton,^39^ who also unknowingly had separate pedigrees that linked to this original one. Within two generations in Australia, the rate of adult males losing vision has dropped from 75% to below 15% and has remained around that level (Table 6). The early publications of the pedigree suggested higher rates of visual loss as many asymptomatic men were missing from the data.

**Table 6:** Falling rate of vision loss among males from the largest Australian LHON pedigree, TAS01

**Generation with earliest year of male birth**	**This study (full genealogical ascertainment)**	**Mackey and Howell**^19^	**Hamilton**^39^**“pedigree 33”**	**Hogg**^38^
IV b1804	3 of 4 (75%)	3 of 4 (75%)	3 of 3 (100%)^a^	3 of 3 (100%)^b^
V b1823	4 of 19 (21%)	4 of 17 (24%)	5 of 5 (100%)	3 of 15 (20%)
VI b1844	13 of 105 12%)	12 of 86 (14%)	7 of 18 (39%)	N/A
VII	27 of 202 (13%)	27	7 of 21 (33%)	N/A
VIII	26 of 180 (14%)	22	N/A	N/A
IX	26 of 229 (11%)	21	N/A	N/A
X	11 of 209 (5%)	2	N/A	N/A

Number of individuals with vision loss and penetrance (%) are shown for each generation. N/A, not applicable.

a Two sub-matriarchs missing.

b One missing unaffected man who moved to Melbourne.

## Discussion

### How common is vision loss from LHON?

The clinical follow-up of LHON families in Australia has been carried out since 1915, and 96 LHON families have now been identified, 40 of which were previously published,^15^^,^^22^ comprising in total 620 individuals (462 males and 158 females) affected by vision loss. The minimum prevalence of vision loss from LHON is one in 68,403 in Australia, which compares with the earlier data from North East England of one in 31,055^25^ and more recent data from Denmark of one in 54,000.^40^ Future updates on population-wide studies will enable us to compare whether findings from other well-described LHON pedigrees in the Netherlands and Finland are similar.^16^^,^^41^

### How common are the three main LHON mtDNA mutations?

The three primary LHON mutations accounted for 88.2% of total LHON cases: 64.5% m.11778G>A mutation, 20.3% m.14484T>C mutation, and only 3.4% m.3460G>A mutation. Because some affected individuals had combined mutations, more than 90% are explained by one or more of the three main mutations. This is similar to an earlier study of 159 multigenerational LHON pedigrees from Europe and Australia (including 16 of the Australian pedigrees), where 69% were m.11778G>A, 14% were m.14484T>C, and 13% were m.3460G>A,^35^ and North East England, where 60% were m.11778G>A, 7% were m.14484T>C, and 33% were m.3460G>A.^25^ This is also similar to a large international study—of which 104/1,512 affected individuals were from Australia—where 69% were m.11778G>A, 17% were m.14484T>C, and 13% were m.3460G>A.^18^

### How likely are LHON mtDNA mutation carriers to be affected?

The penetrance for all LHON mutation carriers was 1 in 6 males (17.5%) and 1 in 20 females (5.4%). The rate in males is lower than the 20% overall risk of vision loss previously reported in Australia in 1992, but the rate in females is slightly higher than the 4% reported.^15^ These findings are similar in the Dutch population, where 29% of male and 6% of female LHON mutation carriers were affected,^42^ although more recent data are required for an accurate comparison.

Importantly, the risk of vision loss was lower than the 50% (males) or 15% (females) risk commonly cited in information accessed by family members (web resources), although penetrance was highly variable among individual Australian pedigrees (1%–73%). To minimize the likelihood of including LHON mutation carriers who may yet lose vision, we only included carriers over 25 years old in our analysis. This is a potential limitation of our study because vision loss due to LHON can occur at any time in life, and therefore, our analysis may underestimate the risk of disease expression over the entire lifespan of an individual.

Our study aimed to fully ascertain all at-risk unaffected male adults within pedigrees. The often-quoted 50% risk comes from the early period of pedigree ascertainment where LHON was presumed to be X-linked—prior to its separation from autosomal dominant optic atrophy by Kjer^43^ and prior to recognition of its mitochondrial transmission.^44^ For most of our pedigrees, being able to confidently quote a much lower and more accurate risk figure can help reduce anxiety within the whole family. However, we acknowledge it may be difficult to persuade ophthalmologists and geneticists to stop quoting 50% risk of vision loss among males. With large families, the members should be informed of the actual risk within their own family where possible.

### How likely are women to be affected?

LHON predominantly affects males, and we observed a male to female ratio of 2.92:1 with 158/620 (25%) affected females. This is similar to the latest reported ratio in sporadic LHON cases in Australia of 2.67:1^22^ and lower than the ratio of 5:1 in 291 familial LHON cases reported previously.^15^ However, our earlier report^15^ noted that 31/135 (23%) living blind individuals were female, which is close to 25% in the current paper. This correlates with the 3:1 male to female ratio found in an international audit of LHON,^18^ again noting that 104/1,512 of these affected individuals were from Australia and thus counted in both studies. Interestingly, both this current study (Figure 2A) and the results published by Poincenot (2020)^18^ show the lack of a female early adult peak in rate of vision loss. Women may lose vision at any age, and although other causes of vision loss can occur, LHON should always be considered when an adult woman in a LHON pedigree loses vision.

### Does having a close family member affected (mother or uncle) increase a family member’s risk of losing vision?

A previous analysis of vision loss in offspring of affected women in Australian and Dutch pedigrees suggested a higher rate, notably in females.^37^ In the Dutch population, children of affected mothers had a higher incidence of vision loss (53% male and 23% female offspring) compared to children of unaffected mothers (27% male and 5% female offspring).^42^ In our Australian study, children of affected mothers had a higher incidence of vision loss (37% male and 29.5% female offspring) than the overall incidence of vision loss observed among all LHON mutation carriers (17.5% male and 5.4% female). The increased risk in daughters is of concern, but it is still more likely that the daughter will never lose vision in her lifetime.

Similarly, we observed a higher incidence of vision loss in children of asymptomatic women with at least one affected brother in some pedigrees. Focusing on six large pedigrees, we compared it to the overall incidence of vision loss in each respective pedigree (nephew risk%:family-male-risk%). The nephew risk was higher in four families, TAS01 (16.6%:11.1%), TAS02 (34.9%: 18.8%), NSW01 (22.2%:15.1%), and NSW02 (20.0%:10.5%), but not in two families, NSW04 (15.1%:27.6%) and WAU01 (25%:27.6%). The incidence of vision loss was higher only among nieces of affected men in the NSW04 family, and the incidence of vision loss was not increased among nephews or nieces of affected men in the WAU01 pedigree.

The fall in vision loss among males from the largest LHON pedigree in Australia^19^ could be explained by a reduction in environmental triggers over time or, alternatively, by a dilution of a background genetic risk with subsequent generations. We have noted high- and low-risk branches within this family previously.^45^ A similar decrease in prevalence of vision loss over successive generations has been observed in a large Italian/Brazilian pedigree with the m.11778G>A mutation.^46^

### Finding modifier genes to predict risk of vision loss

Although the proportion of individuals with vision loss was similar between those with the m.11778G>A or m.14484T>C mutations, after adjusting for sex and mtDNA haplogroup, the odds of vision loss were almost half as low among those with the m.14484T>C mutation compared to those with m.11778G>A. Furthermore, it was reported previously that vision loss is more frequent among individuals harboring the m.11778G>A mutation and mtDNA haplogroup J.^8^ We previously reported very low penetrance in family VIC14 with the m.14484T>C mutation on a background of haplogroup H and that the m.14484T>C mutation is under-represented among haplogroup H mtDNAs that carry a LHON mutation.^7^ In the current study, individuals with haplogroup J were estimated to have almost three times the odds of vision loss compared to those with haplotype H. For genetic counseling purposes, determining the haplogroup may help explain a large, low penetrance pedigree.

There is currently a large amount of work being conducted on the genetics of the optic nerve structure and glaucoma, which may identify some overlapping genetic risk factors for LHON as well as potential new neuroprotective treatments,^47^ and potential biomarkers in LHON are under investigation.^13^ Nuclear genetic modifiers that may also influence LHON penetrance have been identified and require further research.^48^^,^^49^

Many families will often experience stress and anxiety when informed they carry a LHON mutation. If high-risk genetic backgrounds are shown to increase risk of vision loss from LHON, then deciding whether or not to be informed about this risk will be complex given the inability to predict the onset of vision loss and that there are no effective interventions currently. This has been the experience in Huntington disease (HD), which suggests that 80% of those at risk for HD choose not to undergo a predictive genetic test.^50^ Thus, genetic counseling for those affected by vision loss from LHON and their families is imperative.

### Conclusion

We have established and maintain an Australian network of researchers, clinicians, and the LHON community that has guaranteed that families with a known or putative LHON diagnosis have been brought to our attention since 1990. Our study represents, if not all, at least the overwhelming majority of LHON families in Australia. Accurate knowledge of risk is essential for genetic counseling within individual pedigrees and to provide access to existing and experimental treatments, such as idebenone or gene therapy, to prevent vision loss in individuals at particularly high risk. Accurate assessment of risk of vision loss will also be important in guiding families to assisted reproductive technologies such as mitochondrial donation. This knowledge could also assist in the detection and validation of potential biomarkers in LHON and may also inform clinical trial design, as an overestimated risk may lead to an underpowered study or a false claim of efficacy.

## Data and code availability

The published article includes all datasets generated or analyzed during this study.

## Web resources

Australian bureau of statistics, https://abs.gov.au/
LHON information (UK), lhonsociety.org
LHON information (USA), lhon.org

## Supplemental information

Document S1. Figure S1, Tables S1–S6, and supplemental subjects and methods

Document S2. Article plus supplemental information
