# Supplementary material for: Establishing risk of vision loss in Leber hereditary optic neuropathy
Source: Am J Hum Genet. 2021 Oct 19;108(11):2159–70. doi: 10.1016/j.ajhg.2021.09.015 (PMC8595929; doi:10.1016/j.ajhg.2021.09.015)
Supplement: Document S1. Figure S1, Tables S1–S6, and supplemental subjects and methods [file mmc1.pdf]

The American Journal of Human Genetics, Volume *108*

Supplemental information

Establishing risk of vision loss in

Leber hereditary optic neuropathy

M. Isabel G. Lopez Sanchez, Lisa S. Kearns, Sandra E. Staffieri, Linda Clarke, Myra B. McGuinness, Wafaa Meteoukki, Sona Samuel, Jonathan B. Ruddle, Celia Chen, Clare L. Fraser, John Harrison, Alex W. Hewitt, Neil Howell, and David A. Mackey

Supplemental Data

Figure S1. Confirmation of heteroplasmic mutation in individuals from LHON pedigrees NZ10, VIC02 and VIC30 by PCR and Sanger sequencing.

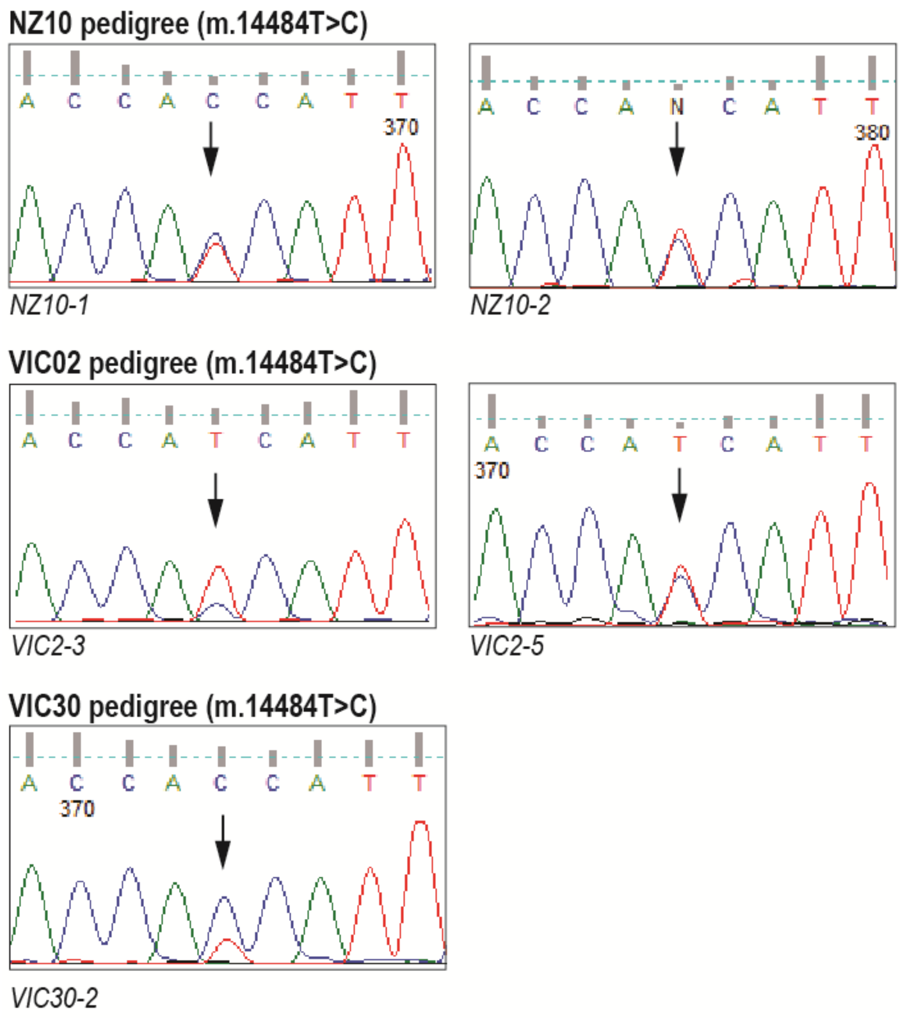

Table S1. Primer sequences of primers used for PCR

Primer	Sequence
11778_Fwd	CCC ACC TTG GCT ATC ATC
11778_Rev	GGT AAG GCG AGG TTA GCG
14484_Fwd	GCA TAA TTA AAC TTT ACT TC
14484_ Rev	AGA ATA TTG AGG CGC CAT TG
3460_Fwd	TTC AAA TTC CTC CCT GTA CG
3460_Rev	GGC TAC TGC TCG CAG TG
DloopHV1_Fwd	TGG GGA AGC AGA TTT GGG TA
DloopHV1_Rev	ACG TGT GGG CTA TTT AGG CT
DloopHV2_Fwd	ATG GGC GGG GGT TGT ATT G
DloopHV2_Rev	CAC AGG TCT ATC ACC CTA TTA ACC

Table S2. Complete mtDNA Sequences of LHON Pedigrees

Sequences are presented as differences from the mtDNA revised Cambridge Reference Sequence (NC_012920.1). Expansions or contractions of simple repeat sequences in the mitochondrial genome are not shown. In addition to the pedigree, we specify the pathogenic LHON mutation and the mtDNA haplogroup according to PhyloTree[1].

Pedigree (mutation)	Haplogroup	mtDNA control region	mtDNA coding region
NSW01 (m.11778G>A)	H1n	146 T:C; 263 A:G; 16468 T:C; 16519 T:C	750 A:G; 1438 A:G; 2098 G:A; 3010 G:A; 4769 A:G; 8860 A:G; 14199 T:C; 15326 A:G; 15742 C:A; 15924 A:G
NSW03 (m.11778G>A)	U5a1	73 A:G; 263 A:G; 16256 C:T; 16270 C:T; 16293 A:G; 16399 A:G	750 A:G; 1438 A:G; 2706 A:G; 3197 T:C; 4769 A:G; 7028 C:T; 7792 C:T; 8860 A:G; 9477 G:A; 11467 A:G; 11719 G:A; 11914 G:A; 12308 A:G; 12372 G:A; 13617 T:C; 14766 C:T; 14793 A:G; 15218 A:G; 15317 G:A; 15326 A:G
NSW04 (m.11778G>A)	J1c1	73 A:G; 185 G:A; 228 G:A; 263 A:G; 295 C:T; 462 C:T; 482 T:C; 489 T:C; 16069 C:T; 16126 T:C	750 A:G; 1438 A:G; 2706 A:G; 3010 G:A; 3394 T:C; 4216 T:C; 4769 A:G; 7028 C:T; 8020 G:A; 8860 A:G; 10398 A:G; 11251 A:G; 11287 T:C; 11719 G:A; 12612 A:G; 13708 G:A; 14766 C:T; 14798 T:C; 15326 A:G; 15452 C:A
NSW06 (m.11778G>A)	H4a1	263 A:G	750 A:G; 1438 A:G; 3992 C:T; 4024 A:G; 4769 A:G; 5004 T:C; 8269 G:A; 8860 A:G; 9123 G:A; 10124 T:C; 14365 C:T; 14582 A:G; 14956 T:C; 15326 A:G
NSW09 (m.11778G>A)	J1c4	73 A:G; 185 G:A; 228 G:A; 263 A:G; 295 C:T; 462 C:T; 489 T:C; 16069 C:T; 16126 T:C	750 A:G; 1438 A:G; 2706 A:G; 3010 G:A; 4216 T:C; 4769 A:G; 7028 C:T; 7853 G:A; 8860 G:A; 9120 A:G; 9632 A:G; 9701 T:C; 10398 A:G; 11251 A:G; 11719 G:A; 12083 T:G; 12612 A:G; 13708 G:A; 14766 C:T; 14798 T:C; 15326 A:G; 15452 C:A
NSW11 (m.11778G>A)	Y2a	73 A:G; 263 A:G; 482 T:C; 16126 T:C; 16231 T:C; 16305 A:G; 16311 T:C; 16362 T:C	750 A:G; 1438 A:G; 2706 A:G; 4769 A:G; 5147 G:A; 5417 G:A; 6941 T:C; 7028 C:T; 7632 T:C; 7859 G:A; 8392 G:A; 8860 A:G; 10398 A:G; 11299 T:C; 11719 G:A; 12161 T:C; 12705 C:T; 14178 T:C; 14693 A:G; 14766 C:T; 14914 A:G; 15244 A:G; 15326 A:G
NSW17 (m.11778G>A)	T2f1	73 A:G; 195 T:C; 263 A:G; 16126 T:C; 16189 T:C; 16294 C:T; 16296 C:T; 16298 T:C; 16519 T:C	709 G:A; 750 A:G; 1438 A:G; 1888 G:A; 2706 A:G; 4216 T:C; 4769 A:G; 4917 A:G; 5277 T:C; 5426 T:C; 6489 C:A; 7028 C:T; 8270 C:T; 8697 G:A; 8860 A:G; 10463 T:C; 11251 A:G; 11719 G:A; 11812 A:G; 13368 G:A; 14233 A:G; 14323 G:A; 14766 C:T; 14905 G:A; 15043 G:A; 15326 A:G; 15452 C:A; 15607 A:G; 15928 G:A
NSW18 (m.11778G>A)	J1c1	73 A:G; 263 A:G; 295 C:T; 462 C:T; 482 T:C; 489 T:C; 16069 C:T; 16126 T:C	750 A:G; 1438 A:G; 2706 A:G; 3010 G:A; 3394 T:C; 4216 T:C; 4769 A:G; 7028 C:T; 8860 A:G; 10398 A:G; 11251 A:G; 11719 G:A; 12612 A:G; 13194 G:A;

			13708 G:A; 14766 C:T; 14798 T:C; 15326 A:G; 15452 C:A
NZ02 (m.11778G>A)	U5b2a1	73 A:G; 150 C:T; 152 T:C; 263 A:G; 16189 T:C; 16325 T:C	750 A:G; 1438 A:G; 1721 C:T; 2706 A:G; 3197T:C; 4732 A:G; 4769 A:G; 6158 A:G; 7028 C:T; 7768 A:G; 8860 A:G; 9477 G:A; 11467 A:G; 11719 G:A; 12308 A:G; 12372 G:A; 13617 T:C; 13637 A:G; 14182 T:C; 14323 G:A; 14766 C:T; 15326 A:G
QLD02 (m.11778G>A)	J2b1	73 A:G; 150 C:T; 152 T:C; 263 A:G; 295 C:T; 489 T:C; 16069 C:T; 16126 T:C; 16193 C:T	750 A:G; 1438 A:G; 2706 A:G; 2789 C:T; 4216 T:C; 4769 A:G; 5633 C:T; 7028 C:T; 7476 C:T; 8860 A:G; 9872 A:G; 10172 G:A; 10398 A:G; 11251 A:G; 11719 G:A; 12612 A:G; 13708 G:A; 13821 C:T; 14766 C:T; 15257 G:A; 15326 A:G; 15452 C:A; 15812 G:A
QLD03 (m.11778G>A)	J1c2	73 A:G; 185 G:A; 188 A:G; 228 G:A; 263 A:G; 295 C:T; 462 C:T; 489 T:C; 16069 C:T; 16126T:C	750 A:G; 1438 A:G; 2706 A:G; 3010 G:A; 4216 T:C; 4769 A:G; 6269 A:C; 7028 C:T; 8860 A:G; 10398 A:G; 11251 A:G; 11719 G:A; 12612 A:G; 13708 G:A; 14766 C:T; 14798 T:C; 15326 A:G; 15452 C:A
QLD04 (m.11778G>A)	T2b4	73 A:G; 263 A:G; 16126 T:C; 16294 C:T; 16304 T:C; 16519 T:C	709 G:A; 750 A:G; 930 G:A; 1438 A:G; 1888 G:A; 2706 A:G; 4216 T:C; 4769 A:G; 4917 A:G; 7028 C:T; 8697 G:A; 8860 A:G; 9254 A:G; 10463 T:C; 11251 A:G; 11719 G:A; 11812 A:G; 13368 G:A; 14233 A:G; 14766 C:T; 14905 G:A; 15326 A:G; 15452 C:A; 15607 A:G; 15928 G:A
TAS01[a] (m.11778G>A)	H	152 T:C; 263 A:G; 16093 T:C	750 A:G; 1438 A:G; 4626 A:G; 4769 A:G; 8860 A:G; 11788 C:T; 15317 G:A; 15326 A:G
TAS05 (m.11778G>A)	H3f	93 A:G; 263 A:G; 16153 G:A; 16311 T:C; 16519 T:C	750 A:G; 1438 A:G; 4769 A:G; 6776 T:C; 8860 A:G; 12811 T:C; 15326 A:G
VIC04 (m.11778G>A)	H1c3	195 T:C; 257 A:G; 263 A:G; 477 T:C; 16290 C:T; 16519 T:C	750 A:G; 1438 A:G; 3010 G:A; 4769 A:G; 8473 T:C; 8860 A:G; 15326 A:G
VIC05 (m.11778G>A)	K2a4	73 A:G; 146 T:C; 152 T:C; 263 A:G; 16129 G:A; 16224 T:C; 16311 T:C; 16519 T:C	709 G:A; 750 A:G; 1438 A:G; 1811 A:G; 2706 A:G; 3480 A:G; 4561 T:C; 4769 A:G; 7028 C:T; 8860 A:G; 9055 G:A; 9698 T:C; 9716 T:C; 10550 A:G; 11299 T:C; 11467 A:G; 11549 C:T; 11719 G:A; 12308 A:G; 12372 G:A; 14167 C:T; 14766 C:T; 14798 T:C; 15326 A:G
VIC06 (m.11778G>A)	H1	152 T:C; 263 A:G; 16189 T:C; 16239 C:T; 16519 T:C	750 A:G; 1438 A:G; 3010 G:A; 4769 A:G; 8860 A:G; 11596 A:G; 13230 C:T; 13768 T:C; 15326 A:G
VIC07 (m.11778G>A)	I2a	73 A:G; 152 T:C; 199 T:C; 204 T:C; 207 G:A; 250 T:C; 263 A:G; 16129 G:A; 16145 G:A; 16223 C:T; 16391 G:A; 16519 T:C	750 A:G; 1438 A:G; 1719 G:A; 2706 A:G; 4529 A:T; 4769 A:G; 7028 C:T; 7813 C:T; 8251 G:A; 8860 A:G; 9266 G:A; 10034 T:C; 10238 T:C; 10398 A:G; 11065 A:G; 11719 G:A; 12501 G:A; 12705 C:T; 13780 A:G; 14766 C:T; 15043 G:A; 15326 A:G; 15758 A:G; 15924 A:G; 15937 A:G
VIC10 (m.11778G>A)	J2a1a	73 A:G; 150 C:T; 152 T:C; 195 T:C 198 C:T; 222 C:T; 263 A:G; 295	750 A:G; 1438 A:G; 1587 T:C; 2706 A:G; 4216 T:C; 4769 A:G; 7028 C:T; 7476 C:T; 7789 G:A; 8860 A:G; 10398

		C:T; 319 T:C; 489 T:C; 513 G:A; 16069 C:T; 16126 T:C; 16145 G:A; 16189 T:C; 16231 T:C; 16261 C:T	A:G; 10499 A:G; 11251 A:G; 11377 G:A; 11581 A:T; 11719 G:A; 12279 A:G[c]; 12612 A:G; 13708 A:G; 13722 A:G; 14133 A:G; 14766 C:T; 15257 G:A; 15326 A:G; 15452 C:A
VIC17 (m.11778G>A)	J1c5a	73 A:G; 150 C:T; 185 G:A; 228 G:A; 263 A:G; 295 C:T; 462 C:T; 489 T:C; 16069 C:T; 16126 T:C; 16519 T:C	750 A:G; 1438 A:G; 2387 T:C; 2706 A:G; 3010 G:A; 4216 T:C; 4769 A:G; 5198 A:G; 7028 C:T; 8860 A:G; 10192 C:T; 10398 A:G; 11251 A:G; 11719 G:A; 12612 A:G; 13708 G:A; 14766 C:T; 14798 T:C; 15326 A:G; 15452 C:A
VIC18 (m.11778G>A)	U4c1	73 A:G; 195 T:C; 263 A:G; 489 T:C; 499 G:A; 16169 C:T; 16179 C:T; 16234 C:T; 16356 T:C; 16519 T:C	750 A:G; 1438 A:G; 1811 A:G; 2706 A:G; 4646 T:C; 4769 A:G; 4811 A:G; 5999 T:C; 6047 A:G; 6146 A:G; 7028 C:T; 8860 A:G; 9070 T:G; 10907 T:C; 11009 T:C; 11332 C:T; 11467 A:G; 11719 G:A; 12308 A:G; 12372 G:A; 14620 C:T; 14766 C:T; 14866 C:T; 15326 A:G; 15693 T:C
VIC19 (m.11778G>A)	B4b1a2	73 A:G; 207 G:A; 263 A:G; 499 G:A; 16136 T:C; 16153 G:A; 16189 T:C; 16217 T:C; 16235 A:G; 16519 T:C	750 A:G; 827 A:G; 1438 A:G; 1664 G:A; 2706 A:G; 4769 A:G; 4820 G:A; 6023 G:A; 6216 T:C; 6413 T:C; 7028 C:T; 8860 A:G; 11719 G:A; 13590 G:A; 14766 C:T; 15326 A:G; 15535 C:T
VIC21 (m.11778G>A)	H13a1a	152 T:C; 263 A:G; 16311 T:C	750 A:G; 1438 A:G; 2259 A:G; 3591 G:T; 4745 A:G; 4769 A:G; 8860 A:G; 9233 T:C; 11410 T:C; 13680 C:T; 14872 C:T; 15326 A:G
VIC22 (m.11778G>A)	H13a1a	263 A:G	750 A:G; 1438 A:G; 2259 A:G; 4745 A:G; 4769 A:G; 8860 A:G; 13680 C:T; 14872 C:T; 15326 A:G; 15355 G:A
VIC25 (m.11778G>A)	T1a1	73 A:G; 152 T:C; 195 T:C; 263 A:G; 16126 T:C; 16163 A:G; 16172 T:C; 16186 C:T; 16189 T:C; 16294 C:T; 16304 T:C; 16519 T:C	709 G:A; 750 A:G; 1438 A:G; 1888 G:A; 2706 A:G; 4216 T:C; 4769 A:G; 4917 A:G; 7028 C:T; 7269 G:A; 8967 G:A; 8860 A:G; 9443 T:C; 9899 T:C; 10463 T:C; 10646 G:A; 11251 A:G; 11719 G:A; 12432 C:T; 12633 C:A; 13368 G:A; 14766 C:T; 14905 G:A;15326 A:G; 15452 C:A; 15607 A:G; 15928 G:A
VIC29 (m.11778G>A)	U5a1a1	73 A:G; 263 A:G; 16256 C:T; 16270 C:T; 16399 A:G	750 A:G; 1438 A:G; 1700 T:C; 2706 A:G; 3197 T:C; 4769 A:G; 5495 T:C; 6905 A:G; 7028 C:T; 8860 A:G; 9477 G:A; 11467 A:G; 11719 G:A; 12308 A:G; 12372 G:A; 13015 T:C; 13617 T:C; 14599 A:G; 14766 C:T; 14793 A:G; 15218 A:G; 15326 A:G; 15924 A:G
WAU03 (m.11778G>A)	I2a	73 A:G; 152 T:C; 199 T:C; 204 T:C; 207 G:A; 250 T:C; 263 A:G; 16126 T:C; 16145 G:A; 16223 C:T; 16391 G:A; 16519 T:C	750 A:G; 1438 A:G; 1719 G:A; 2706 A:G; 3398 T:C; 4529 A:T; 4769 A:G; 7028 C:T; 8251 G:A; 8860 A:G; 10034 T:C; 10238 T:C; 10398 A:G; 11065 A:G; 11719 G:A; 12501 G:A; 12705 C:T; 13780 A:G; 14766 C:T; 15043 G:A; 15326 A:G; 15758 A:G; 15924 A:G
WAU04 (m.11778G>A)	H3	263 A:G; 16189 T:C; 16519 T:C	750 A:G; 1438 A:G; 4769 A:G; 5087 T:C; 6776 T:C; 8860 A:G; 10325 G:A; 15326 A:G
NSW12 (m.14484T>C)	K1c2	73 A:G; 146 T:C; 152 T:C; 263 A:G; 16224 T:C; 16311 T:C; 16320	750 A:G; 1189 T:C; 1438 A:G; 1811 A:G; 2706 A:G; 3480 A:G; 4769 A:G; 7028 C:T; 8860 A:G; 9006 A:G; 9055 G:A; 9698 T:C; 10398 A:G; 10550 A:G;

		C:T; 16356 T:C; 16519 T:C	11299 T:C; 11467 A:G; 11719 G:A; 12308 A:G; 12372 G:A; 14002 A:G; 14040 G:A; 14167 C:T; 14766 C:T; 14798 T:C; 15326 A:G
NSW15 (m.14484T>C)	U5a1a1	73 A:G; 263 A:G; 16256 C:T; 16270 C:T; 16399 A:G	750 A:G; 979 C:T; 1438 A:G; 1700 T:C; 2706 A:G; 3197 T:C; 4769 A:G; 5495 T:C; 6164 C:T; 7028 C:T; 8860 A:G; 9477 G:A; 11467 A:G; 11719 G:A; 12308 A:G; 12372 G:A; 13617 T:C; 14766 C:T; 14793 A:G; 15218 A:G; 15326 A:G; 15924 A:G
NSW19 (m.14484T>C)	L1b1a1	73 A:G; 152 T:C; 182 C:T; 185 G:T; 195 T:C; 247 G:A; 263 A:G; 264 C:T; 357 A:G; 16114 C:T; 16126 T:C; 16187 C:T; 16189 T:C; 16215 A:C; 16223 C:T; 16264 C:T; 16270 C:T; 16278 C:T; 16293 A:G; 16311 T:C; 16519 T:C	709 G:A; 710 T:C; 750 A:G; 769 G:A; 825 T:A; 1018 G:A; 1738 T:C; 2352 T:C; 2706 A:G; 2758 G:A; 2768 A:G; 2885 T:C; 3308 T:C; 3396 T:C; 3594 C:T; 3666 G:A; 3693 G:A; 4104 A:G; 4769 A:G; 5036 A:G; 5046 G:A; 5393 T:C; 5655 T:C; 6548 C:T; 6827 T:C; 6989 A:G; 7028 C:T; 7055 A:G; 7146 A:G; 7256 C:T; 7389 T:C; 7521 G:A; 7867 C:T; 8248 A:G; 8468 C:T; 8655 C:T; 8701 A:G; 8860 A:G; 9540 T:C; 10398 A:G; 10688 G:A; 10810 T:C; 10873 T:C; 11719 G:A; 12519 T:C; 12705 C:T; 13105 A:G; 13506 C:T; 13650 C:T; 13789 T:C; 13880 C:A; 14178 T:C; 14203 A:G; 14560 G:A; 14766 C:T; 14769 A:G; 15115 T:C; 15326 A:G
TAS02 (m.14484T>C)	J1c2c	73 A:G; 146 T:C; 185 G:A; 188 A:G; 222 C:T; 228 G:A; 263 A:G; 295 C:T; 462 C:T; 489 T:C; 16069 C:T; 16126 T:C; 16261 C:T; 16519 T:C	750 A:G; 1438 A:G; 2706 A:G; 3010 G:A; 4216 T:C; 4769 A:G; 7028 C:T; 8610 T:C; 8860 A:G; 10398 A:G; 10685 G:A; 11251 A:G; 11719 A:G; 12612 A:G; 13281 T:C; 13708 G:A; 13933 A:G; 14766 C:T; 14798 T:C; 15326 A:G; 15452 C:A
VIC02[b] (m.14484T>C)	J1b1a	73 A:G; 185 G:A; 242 C:T; 263 A:G; 295 C:T; 462 C:T; 489 T:C; 16069 C:T; 16126 T:C; 16145 G:A; 16172 T:C; 16222 C:T; 16261 C:T	750 A:G; 1438 A:G; 1462 G:A; 2158 A:G; 2706 A:G; 3010 G:A; 4216T:C; 4659 G:A; 4769 A:G; 5460 C:T; 6345 T:C; 7028 G:A; 7299 A:G; 8269 G:A; 8557 G:A; 8860 A:G;10398 A:G; 11251 A:G; 11719 A:G; 12007 G:A; 12612 A:G; 13708 G:A; 13879 T:C; 14766 C:T; 15326 T:C; 15452 C:A
VIC08 (m.14484T>C)	J1c1	73 A:G; 152 T:C; 263 A:G; 295 C:T; 462 C:T; 489 T:C; 16069 C:T; 16126 T:C; 16193 C:T; 16519 T:C	750 A:G; 1438 A:G; 2706 A:G; 3010 G:A; 4216 T:C; 4769 A:G; 7028 C:T; 7789 G:A; 7963 A:G; 8779 C:T; 8860 A:G; 9041 A:G; 10398 A:G; 11251 A:G; 11719 G:A; 12612 A:G; 12681 T:C; 13708 G:A; 14766 C:T; 15326 A:G; 15452 C:A
VIC11 (m.14484T>C)	J1c1	73 A:G; 185 G:A; 189 A:G; 228 G:A; 263 A:G; 295 C:T; 462 C:T; 489 T:C; 16069 C:T; 16126 T:C; 16293 A:G	750 A:G; 1438 A:G; 2706 A:G; 3010 G:A; 3394 T:C; 4216 T:C; 4769 A:G; 7028 C:T; 8860 A:G; 10398 A:G; 11251 A:G; 11719 G:A; 12612 A:G; 13708 G:A; 14766 C:T; 14798 T:C; 15184 T:C; 15326 A:G; 15452 C:A
VIC14 (m.14484T>C)	H3c	195 T:C; 263 A:G; 16176 C:T	750 A:G; 1211 G:A; 1438 A:G; 4769 A:G; 6261 G:A; 6776 T:C; 7444 G:A; 8347 A:G; 8860 A:G; 12957 T:C; 14305 G:A15326 A:G

TAS03 (m.3460G>A)	H5a1	263 A:G; 456 C:T; 16304 T:C	750 A:G; 1438 A:G; 4336 T:C; 4769 A:G; 8860 A:G; 12696 T:C; 15326 A:G; 15833 C:T
VIC01 (m.3460G>A)	H	93 A:G; 207 G:A; 263 A:G; 16519 T:C	750 A:G; 1438 A:G; 4769 A:G; 8860 A:G; 9612 G:A; 11560 A:G; 15058 C:T; 15326 A:G
WAU06 (m.3460G>A)	M53	73 A:G; 240 A:G; 263 A:G; 390 A:T; 438 C:T; 489 T:C; 16051 A:G; 16093 T:C; 16223 C:T; 16316 A:G; 16400 C:T; 16519 T:C	593 T:C; 750 A:G; 2010 T:C; 2706 A:G; 4769 A:G; 5493 T:C; 5821 G:A; 6216 T:C; 6719 T:C; 7028 C:T; 7805 G:A; 8701 A:G; 8860 A:G; 9302 C:T; 9540 T:C; 10084 T:C; 10398 A:G; 10400 C:T; 10873 T:C; 11167 A:G; 11560 A:G; 11719 G:A; 12630 G:A; 12705 C:T; 14766 C:T; 14783 T:C; 15043 G:A; 15301 G:A; 15315 C:T; 15326 A:G
NSW08 (m.14482C>G)	I1a	73 A:G; 199 T:C; 204 T:C; 250 T:C; 263 A:G; 16129 G:A; 16172 T:C; 16223 C:T; 16311 T:C; 16391 G:A; 16519 T:C	750 A:G; 1438 A:G; 1531 C:T; 1719 G:A; 2706 A:G; 3447 A:G; 4529 A:T; 4769 A:G; 6734 G:A; 7028 C:T; 8248 A:G; 8251 G:A; 8616 G:T; 8860 A:G; 9966 G:A; 10034 T:C; 10238 T:C; 10398 A:G; 10550 A:G; 11719 G:A; 12501 G:A; 12705 C:T; 12864 T:C; 13780 A:G; 14766 C:T; 15043 G:A; 15326 A:G; 15589 C:T
VIC20 (m.11778G>A/ m.14484T>C)	U5a1a1	73 A:G; 152 T:C; 263 A:G; 16231 T:C; 16256 C:T; 16270 C:T; 16399 A:G	750 A:G; 1438 A:G; 1700 T:C; 2706 A:G; 3197 T:C; 4769 A:G; 5495 T:C; 7028 C:T; 8860 A:G; 9477 G:A; 11467 A:G; 11719 G:A; 12308 A:G; 12372 G:A; 12771 G:A;13617 T:C; 14766 C:T; 14793 A:G; 15218 A:G; 15326 A:G; 15924 A:G
QLD01 (m.14484T>C/ m.4160T>C)	U4a1a	73 A:G; 152 T:C; 263 A:G; 499 G:A; 16134 C:T; 16356 T:C; 16519 T:C	750 A:G; 961 T:C; 1438 A:G; 1811 A:G; 2706 A:G; 4646 T:C; 4769 A:G; 5999 T:C; 6047 A:G; 6845 C:T; 7028 C:T; 8818 C:T; 8860 A:G; 11332 C:T; 11467 A:G; 11719 G:A; 12308 A:G; 12372 G:A; 12937 A:G; 14620 C:T; 14766 C:T; 15326 A:G; 15693 T:C

[a] Nine members of the TAS01 pedigree were used for sequencing; five of them were sequenced in triplicate. Within the limits of MitoChip-based sequencing, all sequences matched those obtained previously by both manual and automated sequencing procedures.
[b] Sequence change is heteroplasmic in the sample analyzed.

Table S3. Partial mtDNA Sequences of LHON Pedigrees.

Sequences are presented as differences from the mtDNA revised Cambridge Reference Sequence (NC_012920.1). Expansions or contractions of simple repeat sequences in the mitochondrial genome are not shown. In addition to the pedigree, we specify the pathogenic LHON mutation and the mtDNA haplogroup according to MitoMaster[2].

Pedigree (mutation)	Haplogroup	mtDNA control region	mtDNA coding region
NSW02 (m.11778G>A)	H	152 T:C; 263 A:G; 16189 T:C; 16239 C:T; 16519 T:C	4769 A:G; 13768 T:C
NSW23 (m.11778G>A)	J	73 A:G; 150 C:T; 152 T:C; 195 T:C; 215 A:G; 263 A:G; 295 C:T; 319 T:C; 489 T:C; 513 G:A; 16069 C:T; 16126 T:C; 16145 G:A; 16231 T:C; 16261 C:T	3447 A:G; 4216 T:C; 4769 A:G; 7419 G:A; 7476 C:T; 11251 A:G; 11719 G:A; 12612 A:G; 13708 G:A; 13722 A:G; 15257 G:A
NSW26 (m.11778G>A)	K	73 A:G; 146 T:C; 152 T:C; 263 A:G; 16224 T:C; 16259 C:T; 16311 T:C; 16320 C:T; 16519 T:C	3480 A:G; 11299 T:C; 11467 A:G; 11719 G:A
NZ01 (m.11778G>A)	U	73 A:G; 152 T:C; 217 T:C; 263 A:G; 508 A:G; 16051 A:G; 16092 T:C; 16129 G:C; 16189 T:C; 16362 T:C; 16519 T:C	11719 G:A
NZ03 (m.11778G>A)	J	73 A:G; 185 G:A; 188 A:G; 228 G:A; 263 A:G; 295 C:T; 462 C:T; 489 T:C; 16069 C:T; 16126 T:C; 16325 T:C; 16519 T:C	4216 T:C; 4769 A:G; 11719 G:A; 11911 T:C; 12612 A:G; 13708 G:A; 14798 T:C
NZ12 (m.11778G>A)	H	74 T:A; 263 A:G; 315 C:CC; 16337 C:A; 16519 T:C	
QLD05 (m.11778G>A)	HV	263 A:G; 16111 C:T; 16519 T:C	
QLD10 (m.11778G>A)	N	73 A:G; 204 T:C; 263 A:G; 16223 C:T	
QLD11 (m.11778G>A)	W	73 A:G; 195 T:C; 204 T:C; 207 G:A; 263 A:G; 16223 C:T; 16292 C:T	3505 A:G; 11719 G:A
QLD12 (m.11778G>A)	U	195 T:C; 263 A:G; 16192 C:T; 16256 C:T; 16270 C:T; 16320 C:T; 16399 A:G	11467 A:G
VIC03 (m.11778G>A)	U	16192 C:T; 16249 T:C; 16270 C:T; 16311 T:C; 16411 C:G; 16412 G:T	11440 G:A; 11467 A:G; 11719 G:A
VIC31 (m.11778G>A)	J	16069 C:T; 16126 T:C; 16311 T:C; 16411 C:G; 16412 G:T	
VIC41 (m.11778G>A)	H	152 T:C; 263 A:G; 16093 T:C	11719 G:A
VIC43 (m.11778G>A)	K	73 A:G; 114 C:T; 263 A:G; 315 C:CC; 16224 T:C; 16234 C:T; 16311 T:C	11299 T:C; 11467 A:G; 11470 A:G; 11719 G:A
WAU01 (m.11778G>A)	J	73 A:G; 228 G:A; 263 A:G; 295 C:T; 462 C:T; 482 T:C; 489 T:C; 16069 C:T	3394 T:C; 4216 T:C; 4769 A:G; 11251 A:G; 11719 G:A; 12612 A:G; 13708 G:A; 13899 T:C; 14798 T:C

WAU02 (m.11778G>A)	K	73 A:G; 150 C:T; 263 A:G; 315 C:CC; 16224 T:C; 16311 T:C; 16519 T:C	T11299C, A11467G, T11485C, G11719A, C11840T
ACT01 (m.14484T>C)	J	73 A:G; 185 G:A; 228 G:A; 263 A:G; 295 C:T; 462 C:T; 482 T:C; 489 T:C; 16069 C:T; 16126 T:C; 16213 T:C	3394 T:C; 4216 T:C; 4769 A:G; 9682 T:C; 11251 A:G; 11719 G:A; 12612 A:G; 13708 G:A
NZ10[a] (m.14484T>C)	J	150 C:T; 152 T:C; 195 T:C; 215 A:G; 295 C:T; 319 T:C; 16126 T:C; 16145 G:A; 16231 T:C; 16261 C:T	11251 A:G; 11377 G:A
NZ11 (m.14484T>C)	J	150 C:T; 185 G:A; 228 G:A; 295 C:T; 462 C:T; 16126 T:C; 16311 T:C	
SAU05 (m.14484T>C)	H	195 T:C; 263 A:G; 310T:C; 366 G:A; 16448 T:C	11719 G:A
VIC30[a] (m.14484T>C)	H	153 A:G; 195 T:C; 263 A:G	14299 T:A; 14487 T:G; 14488 T:G
VIC42 (m.14484T>C)	J	73 A:G; 185 G:A; 228 G:A; 263 A:G 295 C:T; 309 C:CCT; 310 T:C; 462 C:T; 482 T:C; 16126 T:C; 16213 G:A	14484 T:C; 14766 C:T; 14798 T:C
NSW07 (m.3460G>A)	T	73 A:G; 263 A:G; 331 A:G; 16126 T:C; 16234 T:C; 16294 C:T; 16296 C:T; 16519 T:C	4216 T:C; 4769 A:G; 4917 A:G; 11251 A:G; 11719 G:A; 11812 A:G; 13965 T:C; 13966 A:G; 14687 A:G; 15928 G:A
VIC28 (m.3460G>A)	H	114 C:T; 146 T:C; 195 T:C; 16288 T:C; 16302 A:G; 16362 T:C	
SAU04 (m.4171C>A)	HV0	72 T:C; 263 A:G; 195 T:C; 16497 A:G	

[a] Sequence change is heteroplasmic in the sample analysed.

Table S4. Updated Assignments of Australian LHON Matrilineal Pedigrees

Previous Assignment	Updated Assignment	Reference
NSW13 (m.14484T>C)	NSW19[a]	
NSW21(m.11778G>A)	VIC29[a, b]	
QLD08 (m.14484T>C)	TAS02[b]	
SAU01 (m.11778G>A)	NSW23[b]	Chan *et al*.[3]
TAS04	None *OPA1*[c]	Mackey and Buttery[4]
VIC12	None[d]	
VIC13 (m.11778G>A)	VIC04[b]	
VIC16 (m.11778G>A)	TAS01[a, b]	
VIC23 (m.11778G>A)	TAS01[a, b]	
VIC26 (m.11778G>A)	TAS01[a, b]	
VIC27 (m.11778G>A)	TAS01[b]	
VIC44 (m.14484T>C)	TAS02[b]	

[a] Reassignment was made on the basis of mtDNA sequence analysis.
[b] Confirmed or determined by further genealogical analysis.
[c] Updated diagnosis of autosomal dominant optic atrophy
[d] Determination of the complete mtDNA sequence from one family member failed to reveal a LHON mutation. As a result, no assignment is made pending further investigation.

Table S5. mtDNA haplogroup distribution amongst asymptomatic and affected individuals

mtDNA Haplotype	Mutation, number (%)							Total
	11778	14484	3460	14484/ 4160	14482	14484/ 11778	4171	
	n=3,986	n=1,215	n=176	n=84	n=54	n=41	n=12	n=5,568
H	2,699 (67.7)	98 (8.1)	128 (72.7)				12 (100)	2,937 (52.7)
J	495 (12.4)	934 (76.9)						1,429 (25.7)
U	241 (6.0)	26 (2.1)		84 (100)		41 (100)		392 (7.0)
I	126 (3.2)				54 (100)			180 (3.2)
K	97 (2.4)	67 (5.5)						164 (2.9)
L		54 (4.4)						54 (1.0)
T	29 (0.7)		10 (5.7)					39 (0.7)
B	23 (0.6)							23 (0.4)
M			16 (9.1)					16 (0.3)
N	16 (0.4)							16 (0.3)
Y	14 (0.4)							14 (0.3)
W	7 (0.2)							7 (0.1)
Unknown	239 (6.0)	36 (3.0)	22 (12.5)					297 (5.3)
Vision loss								
Carrier	3,586 (90.0)	1,089 (89.6)	155 (88.1)	23 (27.4)	47 (87.0)	38 (92.7)	10 (83.3)	4,948 (88.9)
Affected	400 (10.0)	126 (10.4)	21 (11.9)	61 (72.6)	7 (13.0)	3 (7.3)	2 (16.7)	620 (11.1)

Table S6. Vision loss in offspring of affected women

	Pedigree_Mother ID	Individuals with vision loss/ Total offspring over 25 years old; [penetrance]	
	m.11778G>A	Male	Female
1	ACT02_210411	0/1	0/1
2	ACT02_210414	1/1	0/0
3	NSW01_177394	0/0	0/2
4	NSW02_173447	0/0	0/2
5	NSW03_107116	0/1	1/2
6	NSW03_107118	0/0	0/2
7	NSW04_177055	1/2	1/1
8	NSW04_177163	0/1	0/1
9	NSW04_177157	1/2	0/0
10	NSW04_177052	1/3	0/0
11	NSW04_177060	2/2	0/1
12	NSW04_177117	0/1	0/2
13	NSW04_210438	2/4	0/6
14	NSW05_177864	1/3	0/1
15	NSW06_177316	0/2	0/1
16	NSW06_177330	0/0	0/1
17	NSW06_177338	0/1	0/1
18	NSW09_107204	0/3	1/2
19	NSW09_107215	1/3	0/2
20	NSW09_107201	1/4	0/2
21	NSW09_107232	0/1	0/0
22	NSW09_107254	1/1	0/0
23	NSW11_177475	1/2	0/1
24	NSW20_107993	2/2	1/1
25	NSW20_107997	1/1	0/0
26	NZ02_178125	1/2	0/0
27	NZ02_178126	1/2	0/1
28	QLD02_108797	0/0	0/1
29	QLD02_108510	0/2	0/7
30	VIC21_108837	0/1	0/3
31	VIC21_107370	0/3	0/2
32	QLD12_197430	0/0	0/1
33	TAS01_105409	1/1	1/1
34	TAS01_104842	0/2	0/0
35	TAS01_105041	1/1	0/4
36	TAS01_105245	0/1	2/2
37	TAS01_104467	1/3	0/7
38	TAS01_105011	2/2	0/2
39	TAS01_105194	0/2	0/1
40	TAS01_105223	0/2	0/2
41	TAS01_105255	0/0	0/3
42	TAS01_174915	3/3	0/1
43	TAS01_174813	0/0	0/2
44	TAS01_174490	0/0	0/2
45	TAS01_174090	0/1	0/1
46	TAS01_174449	1/3	0/4
47	TAS01_174824	0/2	0/0
48	VIC06_109398	1/4	1/5
49	VIC06_109401	0/1	0/1
50	VIC10_156049	0/1	0/0
51	VIC40_178503	1/2	0/1
52	WA01_107453	1/2	0/0
53	WA01_107435	0/0	1/1

54	WA01_107426	0/0	1/3
55	WA02_195793	0/2	0/0
56	WA04_109455	0/1	0/1
	m.14484T>C		
57	NSW19_107852	0/2	0/1
58	SA02_178275	1/2	0/0
59	TAS02_175792	0/0	0/3
60	TAS02_175621	0/1	0/0
61	TAS02_175743	0/1	0/2
62	TAS02_175324	0/0	0/2
63	TAS02_172941	1/2	0/1
64	TAS02_206147	0/0	0/1
65	TAS02_173057	0/6	0/3
66	VIC02_109179	0/0	3/3
67	VIC02_109183	0/1	0/0
68	VIC11_109070	0/1	0/2
69	VIC11_109069	0/0	1/2
70	VIC11_108996	1/3	1/1
	m.3460G>A		
71	VIC01_108975	0/0	1/2
72	NSW07_171660	0/1	0/1
	m.14484T>C + m.4160T>C		
73	QLD01_108448	0/0	1/1
74	QLD01_108424	2/2	1/2
75	QLD01_108446	2/2	1/1
76	QLD01_108491	1/3	1/1
77	QLD01_108444	0/3	0/1
78	QLD01_108437	1/1	1/1
79	QLD01_108463	1/1	2/2
80	QLD01_108495	0/0	1/1
81	QLD01_108474	1/1	0/0
82	QLD01_108469	1/1	4/4
83	QLD01_108473	0/0	2/2
84	QLD01_108421	0/1	1/1
85	QLD01_108439	1/1	0/0
86	QLD01_108412	3/4	4/6
87	QLD01_108441	0/0	2/2
88	QLD01_108451	0/0	1/1
89	QLD01_108454	0/0	1/1
90	QLD01_108456	1/3	0/0
91	QLD01_108466	1/1	2/2
92	QLD01_108488	0/0	1/1
93	QLD01_108493	0/0	1/2
	m.14482C>G		
94	NSW08_108088	1/1	1/2
	Total	49/132 [37.1%]	44/149 [29.5%]

Supplemental Method

Participant recruitment

In 1990, Professor David Mackey (DM) contacted all ophthalmologists in Tasmania and all organisations for the blind in Australia asking to be put in contact with any patient affected by LHON. In addition, DM contacted all clinicians and researchers who had published on LHON pedigrees in Australia. LHON family members were asked to provide their matrilineal family tree as far back as possible, often with assistance of other family members. A professional genealogist then expanded each pedigree using national genealogical resources such as the digitised and microfilmed births deaths and marriage records, published family trees and histories available at genealogical society libraries, as well as legal documents such as wills that name living descendants. Many smaller families were subsequently linked into the main pedigrees [4].

Subsequently, DM contacted every ophthalmologist in Australia and offered DNA testing for patients suspected to be affected by LHON. This identified some new branches of the main families and provided additional updated pedigree information as well as new mainly small families[3]. Since 1995, LHON patients and families have been referred to our clinics in Melbourne, Tasmania and Western Australia by ophthalmologists across Australia, and new patients have also self-referred to our research team or clinics. In addition to genealogical linkage investigations, we use mtDNA sequencing to identify related pedigrees and conduct more exhaustive genealogical searches to confirm potential linkages. We provide national genetic counselling advice to LHON families.

Supplemental References

1. van Oven, M., and Kayser, M. (2009). Updated comprehensive phylogenetic tree of global human mitochondrial DNA variation. Hum Mutat 30, E386-394.
2. Lott, M.T., Leipzig, J.N., Derbeneva, O., Xie, H.M., Chalkia, D., Sarmady, M., Procaccio, V., and Wallace, D.C. (2013). mtDNA Variation and Analysis Using Mitomap and Mitomaster. Curr Protoc Bioinformatics 44, 1.23.21-26.
3. Chan, C., Mackey, D.A., and Byrne, E. (1996). Sporadic Leber hereditary optic neuropathy in Australia and New Zealand. Aust N Z J Ophthalmol 24, 7-14.
4. Mackey, D.A., and Buttery, R.G. (1992). Leber hereditary optic neuropathy in Australia. Aust N Z J Ophthalmol 20, 177-184.
